# Insulin resistance in a cohort of 5–15 year old children in urban Sri Lanka

**DOI:** 10.1186/s13104-017-2658-x

**Published:** 2017-07-28

**Authors:** V. P. Wickramasinghe, C. Arambepola, P. Bandara, M. Abeysekera, S. Kuruppu, P. Dilshan, B. S. Dissanayake

**Affiliations:** 1 Department of Paediatrics, Faculty of Medicine, University of Colombo, Kynsey Road, Colombo, Sri Lanka; 2 Department of Community Medicine, Faculty of Medicine, University of Colombo, Colombo, Sri Lanka

**Keywords:** Insulin resistance, Sri Lankan children, Childhood obesity, Low birth weight

## Abstract

**Background:**

South Asian populations develop insulin resistance from a young age. Poor intrauterine growth and increased rates of post natal growth predisposes to develop insulin resistance later in life. This study identifies insulin resistance and relation to birth weight among a group of 5–15 year old children of urban Sri Lanka.

**Methods:**

A cross sectional descriptive study, using two-stage probability proportionate cluster sampling technique. After a 12 h overnight fast, blood was drawn for fasting blood glucose and insulin. OGTT was performed with 2 h random blood glucose. Basic anthropometry was assessed and insulin resistance measured by HOMA-IR.

**Results:**

Of 309 children (boys 133) 13 (4.2%) were obese and 35 (11.3%) were overweight. Eight had impaired glucose homeostasis but no diabetes mellitus. The mean (SD) fasting insulin was 37.8 (37.9) and 32.5 (40.4) pmol/L in girls and boys respectively. 2 h post glucose insulin in girls and boys were 258 (324) and 152 (168) pmol/L respectively. The mean HOMA-IR was 1.1 (1.1) and 0.94 (1.2) for girls and boys respectively. The 4th quartile value of HOMA-IR for the whole population was 1.2 (95% CI 1.1, 1.3) and in obese children 2.26 (95% CI 2.0, 3.1). Fasting and 2 h insulin and HOMA-IR was not affected by birth weight but showed significant difference when compared across present BMI tertile with significantly high values in the highest tertile.

**Conclusion:**

Although many children were able to control glucose within normal limits, evidence of early development of insulin resistance was seen. Children born small but became obese, had the highest risk of developing insulin resistance.

## Background

Prevalence of childhood obesity is rising all over the world parallel to the economic transition that is taking place in many developing countries. South Asian populations are particularly affected due to its fast economic growth and genetic predisposition, leading to the development of many adverse metabolic consequences including insulin resistance [1].

Insulin resistance is a state where the produced insulin exerts a suboptimal biological action [2], resulting in a decrease in the ability of insulin to stimulate skeletal muscle and adipose tissue for glucose uptake. Insulin resistance leads to an increase flux of free fatty acid following lack of suppression of lipolysis in adipose tissue associated with increase hepatic lipogenesis leading to the accumulation of triglyceride in hepatocytes causing hepatic steatosis [3]. Hepatic steatosis with elevated liver enzymes, especially alanine amino transferase (ALT) is associated with insulin resistance, is known as the hepatic manifestation of metabolic syndrome [2].

Many complications related to obesity such as hypertension [4], dyslipidaemia [5], hepatic steatosis [6] and metabolic syndrome [7] are driven by insulin resistance. Although many consider that cardiovascular metabolic derangements are seen mainly among adults, they are frequently seen among obese adolescents and pre-pubertal children as well [8]. Insulin resistance is seen among children that are overweight and obese and the alarming fact is that it persists into adulthood.

South Asian populations are prone to develop type 2 diabetes mellitus with high insulin resistance more frequently, than many other ethnic groups [9]. In the last few decades, the prevalence of diabetes mellitus has increased while the mean age of onset had decreased. Prevalence of type 2 diabetes, among Sri Lankan adults is about 10.3% [10]. Prevalence of type 2 diabetes mellitus, among younger age groups, is increasing, which represents only the tip of the iceberg and therefore insulin resistance is likely to commence from much younger age. Although metabolic syndrome is not common among children of the general population (1.6%), it is high among obese children (22.1%) [8]. High insulin resistance had been reported among Indian post-pubertal children and was closely association with both intra-abdominal and subcutaneous adiposity [11]. Insulin resistance in Sri Lankan children has not studied before and understanding more about problem would help to identify the age of onset and other factors that would determine the development of insulin resistance, which would help in controlling non-communicable disease in the long run. In this study, we attempt to describe the patterns of insulin resistance in a cohort of 5–15 year old urban children of Sri Lanka and factors that could be contributing to its development.

## Methods

A cross-sectional descriptive study was carried out among 5–15 year old apparently healthy Sri Lankan children living in Colombo, which represents the most urban district in Sri Lanka. Sample for the study was randomly selected (with boys and girls represented in almost equal proportions from each age of 5–15 years) from a larger epidemiological study on screening for metabolic abnormalities among 920 children recruited using a two-stage, probability proportionate to size, cluster sampling technique. Students with any illness during past 2 weeks or on any long term medication medication or having long standing illness, were excluded. Students were explained about the study and those who were willing to participate were identified and their parents were informed about the procedure and written consent from parents and assent from children above 10 years were obtained. The size of the sub sample was determined as 309 based on an expected proportion of insulin resistance of 7.5% precision of 0.05 and non-response of 10% [8]. Every third child from the recruitment register was subsampled for this study and in order to complete the sample size, last seven children were consequently recruited. The Ethics Review Committees of the Faculty of Medicine, University of Colombo and Lady Ridgeway Hospital for Children approved the study.

## Assessment of nutritional status

Height, weight, waist circumference (WC) and hip circumference (HC) were measured in all participants using a standard protocol [12]. Body mass index (BMI) (weight (kg)/height (m)^2^), waist hip ration (WHR) (WC(cm)/HC (cm)) and waist to height ratio (WHtR) (WC (cm)/Ht (cm)) were calculated. Nutritional status of children was assessed based on IOTF [13] and categorized as obese, overweight and lean. Cutoff values for %FM used was 28.6% for boys and 33.7% for girls [14]. All assessments were done by trained research assistants who were medical graduates.

## Assessment of metabolic derangements

Blood pressure was measured in the seated position using a mercury sphygmomanometer after a 10 min rest period. The first and fifth Korotkoff sounds were used to represent the systolic (SBP) and diastolic (DBP) blood pressure, respectively.

Blood was drawn after a 12 h overnight fast by trained nurses for fasting blood glucose (FBG) lipid profile, fasting insulin and alanine amino transferase (ALT). Oral glucose tolerance test (OGTT) was done after giving a standardised dose of anhydrous glucose, 1.75 g/kg per body weight to a maximum of 75 g dissolved in water and given as a drink. Blood was drawn 2 h later for random blood glucose (RBG) and serum insulin.

Fasting insulin, fasting glucose to fasting insulin ratio (FGIR), oral glucose tolerance test (OGTT), quantitative insulin sensitivity check index (QUICKI), HOMA IR and HOMA B were used to assess the insulin resistance in this study sample.

Blood glucose (both FBG and RBG) was assessed by enzymatic spectrophotometric method using glucose oxidase and peroxidase enzymes. Quantitative analysis was done using spectrophotometer (BioSystems^®^). Cholesterol ester molecule was cleaved using cholesterol oxidase and peroidase enzymes and cholesterol level was assessed quantitatively using spectrophotometer (BioSystems^®^). Enzymatic cleavage of triglyceride (TG) was done using glycerol phosphate oxidase and peroxidase enzymes and its end products were assessed quantitatively by spectrophotometer (BioSystems^®^). HDL-cholesterol was measured using enzymatic spectrophotometry with enzymatic analysis using cholesterol esterase, cholesterol oxidase and peroxidase. Quantitative assessments were done by spectrophotometer (Randox^®^). LDL-cholesterol was calculated using, total cholesterol—(HDL + TG/5) equation. Serum insulin was assessed by solid phase, enzyme labelled chemiluminescent immunometric assay (Immulite 1000^®^).

## Definition of metabolic derangements

Metabolic derangements were identified as: WC for age above 90th centile of UK standards [15]; abnormal glucose homeostasis if FBS >5.6 mmol/L or 2 h OGTT value >7.8 mmol/L; HDL <1.03 mmol/L; triglyceride ≥1.7 mmol/L [16]; and blood pressure, >+2SD for age for both SBP and DBP of UK standards [17]. This cutoff value for SBP and DBP was chosen instead of the cutoff values given by IDF definition, 130/85 mmHg, as it would be suitable only for the tallest 15 year old children and it could lead to an under estimation of hypertension.

Insulin resistance was measured by two surrogate measures: fasting hyperinsulinemia and homeostasis model assessment (HOMA) [18]. HOMA was calculated: (fasting insulin (mU/ml) × fasting glucose (mmol/L))/22.5. QUICKI was calculated as 1/(log fasting insulin (U/l) + log fasting glucose (mg/dl)) [19]. FGIR was calculated as fasting insulin (U/l)/fasting glucose (mg/dl) [20]. The homoeostasis model assessment was used to estimate pancreatic β cell function (HOMA-B %) and was calculated as 20 × fasting insulin (U/l)/(fasting glucose (mmol/L)−3.5) [18].

## Data analysis

Data were entered and anlyzed using Number Cruncher Statistical Software (NCSS) for windows. Sample was segregated into two age groups, 5 to <10 years and those who are 10 years and above till completion of 15 years. This is in keeping in line with the IDF categorization of ages for the diagnosis of metabolic syndrome [16].

## Results

A total of 309 children (176 girls) were studied. Age and sex specific anthropometric and metabolic data are given in Table 1. Mean fasting insulin level was higher and almost doubled in the older age group compared to the younger age group of the same sex. The 2 h post glucose load insulin levels were more than two-fold in the older age group and were much higher among girls than in boys. HOMA-IR was doubled in the older age group denoting that insulin sensitivity is decreasing with advance in age. Parameters increased with increasing degree of adiposity and significantly higher levels were seen in older age groups. Mean HOMA-IR levels calculated for the whole group of girls and boys were 1.1 (SD = 1.1) and 0.94 (SD = 1.2), respectively. The 4th quartile value of HOMA-IR for the whole sample was 1.2 (95% CI 1.1, 1.3) and 2.26 (95% CI 2.0, 3.1) in obese children.

**Table 1:** Distribution of metabolic and anthropometric parameters of the whole study population and stratified according to age categories

	5–10 year^#^		10–15 years^#^		Total (5–15 years)	
	Female	Male	Female	Male	Female	Male
N	104	54	72	79	176	133
Age (Yrs)	7.9 ± 1.3^§^	7.9 ± 1.4^§^	12.8 ± 1.7*	12.1 ± 1.3	9.9 ± 2.8	10.4 ± 2.4
BMI (kgm^−2^)	15.9 ± 3.5^§^	15.3 ± 2.8^§^	18.5 ± 4.2*	16.9 ± 3.3	17.0 ± 4.0	16.3 ± 3.2
BMIZ	−0.45 ± 1.7	−0.83 ± 1.7	−0.45 ± 1.7	−0.77 ± 1.8	−0.45 ± 1.71	−0.79 ± 172
FM (kg)	6.7 ± 5.3^§^	5.2 ± 4.4^§^	12.5 ± 7.9*	7.8 ± 5.3	9.1 ± 7.0*	6.7 ± 5.1
Percentage FM	23.5 ± 9.7*^§^	19.2 ± 9.8	28.1 ± 10.7*	19.8 ± 8.9	25.4 ± 10.3*	19.6 ± 9.2
FMI (kgm^−2^)	4.0 ± 2.6*^§^	3.1 ± 2.3	5.5 ± 3.3*	3.6 ± 2.3	4.6 ± 3.0*	0.87 ± 0.05
WC (cm)	56.9 ± 9.1^§^	54.3 ± 7.8^§^	66.7 ± 9.8*	63.2 ± 10.2	60.8 ± 10.5	59.6 ± 10.3
HC (cm)	66.9 ± 9.8*^§^	62.4 ± 8.7^§^	81.4 ± 10.8*	72.4 ± 9.4	72.7 ± 12.4*	68.3 ± 10.3
WHR	0.85 ± 0.04*^§^	0.87 ± 0.04	0.82 ± 0.07*	0.87 ± 0.05	0.84 ± 0.06*	0.87 ± 0.05
WHtR	0.45 ± 0.05*	0.43 ± 0.04	0.45 ± 0.06	0.43 ± 0.06	0.45 ± 0.05*	0.43 ± 0.05
SBP (mmHg)	88.6 ± 10.0^§^	90.2 ± 10.5^§^	101.1 ± 10.3	104.3 ± 11.4	93.6 ± 11.8*	98.6 ± 13.0
DBP (mmHg)	54.2 ± 8.7^§^	56.8 ± 8.0^§^	63.8 ± 8.8	66.4 ± 7.4	58.1 ± 9.9*	62.5 ± 9.1
Fasting blood sugar (mmol/l)	4.3 ± 0.46^§^	4.2 ± 0.6^§^	4.7 ± 0.52	4.6 ± 0.5	4.4 ± 0.5	4.4 ± 0.6
Random blood sugar (2 h OGTT) (mmol/l)	4.9 ± 0.9*^§^	4.5 ± 0.9^§^	5.5 ± 0.9*	4.9 ± 1.0	5.2 ± 0.9*	4.7 ± 1.0
Total cholesterol (mmol/l)	4.7 ± 0.6*	4.5 ± 0.8	5.0 ± 3.1	4.7 ± 0.7	4.8 ± 2.1	4.6 ± 0.8
Triglyceride (mmol/l)	0.8 ± 0.3^§^	0.8 ± 0.3	1.0 ± 0.5*	0.90 ± 0.3	0.9 ± 0.4	0.9 ± 0.3
HDL (mmol/l)	1.2 ± 0.4	1.2 ± 0.3	1.2 ± 0.3	1.2 ± 0.3	1.2 ± 0.4	1.2 ± 0.3
Insulin (fasting) (pmol/l)	29.4 ± 34.8^§^	24.8 ± 16.7	49.8 ± 39.3	37.8 ± 49.5	37.8 ± 37.9	32.5 ± 40.4
Insulin (2 h OGTT) (pmol/l)	149.6 ± 162.9^§^	92.3 ± 67.7^§^	416.7 ± 425*	195.2 ± 201.7	258.0 ± 324.5*	152.1 ± 168.3
HOMA-IR	0.83 ± 1.0^§^	0.66 ± 0.48^§^	1.45 ± 1.1	1.1 ± 1.48	1.1 ± 1.1	0.94 ± 1.2
ALT (IU/l)	15.4 ± 14.6^§^	12.4 ± 14.1	22.1 ± 18.2*	12.8 ± 14.9	18.1 ± 16.4	12.6 ± 14.1

* p < 0.05 when compared between the two sex groups in each age category or whole group

^§^p < 0.05 when compared between the two age groups within each gender

^#^Age categorization is done as 5 year to less than 10 years, and 10 years and more to 15 years

Table 2 shows the mean fasting insulin, 2 h post glucose insulin, QUICKY, FIGR, HOMA-IR and HOMA-B for the whole group stratified by different states of anthropometric and other metabolic parameters. Except for total cholesterol, mean fasting insulin and 2 h post glucose insulin levels were elevated in the group that had deranged metabolic parameters. These data show an association between metabolic derangements and insulin secretion. QUICKI, FGIR and HOMA-B showed more or less a consistent pattern of elevated levels in the deranged component of each parameter, compared to the normal parameter. HOMA-B values were higher in the deranged metabolic parameter compared to the normal level, which is contrary to what was seen among diabetic patients, probably denoting the initial increase in beta cell mass and function before beta cell function is decreased.

**Table 2:** Distribution of mean values of different measures of insulin resistance/sensitivity according to different status of anthropometric and metabolic measures

Anthropometric/metabolic		Measure of insulin resistance/sensitivity					
Parameter	Status	Fasting insulin	2 h post glucose insulin	HOMA_IR	QUICKY	FIGR	HOMA–B^¥^
		35.52 ± 39.0	212.91 ± 273.37	1.02 ± 1.14	0.41 ± 0.05	6.41 ± 7.04	177.86 ± 533.22
BMI^§,†^ (kgm^−2^)	Lean	30.27 ± 34.09	172.53 ± 204.11	0.87 ± 1.01	0.42 ± 4.59	0.54 ± 6.12	161.84 ± 566.44
	Over weight	54.58 ± 32.5	435.68 ± 428.47	1.57 ± 0.96	0.37 ± 4.49	9.80 ± 5.76	225.12 ± 215.56
	Obese	89.54 ± 77.82	437.76 ± 520.54	2.55 ± 2.21	0.35 ± 4.27	0.16 ± 0.14	372.27 ± 401.62
WC^‡^ (cm)	Normal	27.85 ± 33.87	142.20 ± 139.32	0.80 ± 0.99	0.42 ± 4.41	5.06 ± 6.06	157.19 ± 606.81
	High	53.31 ± 44.23*	378.14 ± 407.74*	1.54 ± 1.28*	0.38 ± 4.69*	0.10 ± 8.11*	225.88 ± 296.73
DBP (mmHg)	Normal	33.56 ± 32.52	192.95 ± 222.51	0.96 ± 0.95	0.41 ± 4.81	6.07 ± 5.94	174.75 ± 544.42
	High	63.72 ± 87.98*	499.33 ± 605.00*	1.85 ± 2.56*	0.39 ± 6.10*	0.11 ± 0.16*	222.88 ± 337.87
CHO (mmol/l)	Normal	36.14 ± 42.14	213.36 ± 285.08	1.03 ± 1.21	0.41 ± 4.91	6.58 ± 7.70	200.59 ± 584.77
	High	33.10 ± 26.82	207.61 ± 233.74	0.98 ± 0.87	0.41 ± 4.97	5.80 ± 4.33	104.52 ± 312.75
TG (mmol/l)	Normal	34.26 ± 38.59	200.69 ± 260.97	0.98 ± 1.13	0.41 ± 4.84	6.20 ± 6.98	176.88 ± 543.85
	High	63.89 ± 40.88*	488.73 ± 413.19*	1.90 ± 1.21*	0.36 ± 4.78*	0.11 ± 7.27*	200.89 ± 168.69
HDL (mmol/l)	Normal	30.79 ± 36.05	198.31 ± 264.91	0.89 ± 1.07	0.42 ± 4.61	5.49 ± 6.35	162.85 ± 480.62
	Low	43.71 ± 42.60*	238.33 ± 286.97	1.24 ± 1.23*	0.40 ± 5.24*	8.00 ± 0.08*	203.89 ± 615.34
Metabolic syndrome	Absent	35.33 ± 38.70	212.41 ± 267.37	1.02 ± 1.13	0.41 ± 4.8	6.38 ± 7.00	179.73 ± 540.38
	Present	41.75 ± 50.73	229.53 ± 452.2	1.18 ± 1.49	0.42 ± 6.40	7.60 ± 8.87	115.66 ± 168.51
ALT (IU/l)	Normal	34.09 ± 38.13	202.63 ± 255.14	0.98 ± 1.11	0.41 ± 4.79	6.15 ± 6.85	170.11 ± 433.23
	High	46.31 ± 47.42	324.83 ± 408.88*	1.37 ± 1.46	0.40 ± 5.75	8.17 ± 8.37	378.60 ± 979.31
Presence of number of abnormal metabolic abnormalities^§^	0	25.83 ± 22.53	137.60 ± 123.57	0.75 ± 0.68	0.42 ± 4.22	4.63 ± 3.96	150.98 ± 505.55
	1	39.47 ± 47.61	237.02 ± 259.95	1.13 ± 1.41	0.41 ± 4.96	7.21 ± 8.42	189.48 ± 644.06
	2	58.47 ± 51.17	404.74 ± 475.98	1.68 ± 1.42	0.38 ± 5.02	0.11 ± 0.10	249.12 ± 373.67
	3	93.29 ± 45.84	514.99 ± 421.12	2.84 ± 1.29	0.33 ± 2.72	0.16 ± 8.25	217.72 ± 131.79
	4	130.57	1391.93	3.68	0.32	0.24	417.24
%FM group	Normal	30.70 ± 37.73	160.79 ± 159.89	0.89 ± 1.10	0.42 ± 4.64	5.53 ± 0.07	160.55 ± 573.85
	High	54.71 ± 38.40*	423.07 ± 469.82*	1.56 ± 1.14*	0.38 ± 4.56*	9.92 ± 6.98*	246.84 ± 318.68

There were no children with elevated (>2SD) systolic BP

* p < 0.05; when compared between normal and high value of each parameter assessed

^§^One way ANOVA (Alpha 0.05) is used in the comparison of IR markers across different BMI categories, and across different numbers of metabolic derangements. Fasting insulin, HOMA-IR and FIGR differed from each BMI category. 2 h post glucose insulin and QUICKI the normal BMI category from other 2 BMI categories

^†^BMI categorization is based on IOTF cutoff values

^‡^Based on British centile cutoff values

^¥^HOMA-B did not show significant differences across different BMI categories or number of metabolic derangements

Table 3 shows the correlation between different anthropometric and metabolic markers and measures of insulin resistance. Fasting insulin, 2 h OGTT insulin, HOMA-IR and FIGR showed the best correlations while other measures of insulin resistance showed weaker relationships. Although WC and HC showed significant (p < 0.01) correlation with measures of insulin resistance, WHR did not show a significant correlation. FIGR showed significant (p < 0.01) positive correlation with all anthropometric measures. WHtR showed a significant correlation with most of the measures of insulin resistance. Blood pressure (p < 0.01), triglyceride (p < 0.01) and ALT (p < 0.05) showed strong association with the main measures of insulin resistance and cholesterol did not show an association with any of the measures of insulin resistance.

**Table 3:** Associations between Insulin levels and HOMA-IR with anthropometry and other metabolic parameters

	Fasting insulin	2 h post glucose insulin	HOMA-IR	QUICKI	FIGR	HOMA-B
BMI (kgm^−2^)	0.487*	0.539*	0.486*	−0.578*	0.487*	0.103
FM (kg)	0.470*	0.613*	0.472*	−0.571*	0.469*	0.098
%FM	0.381*	0.489*	0.379*	−0.482	0.383*	0.074
WC (cm)	0.454*	0.563*	0.459*	−0.572*	0.439*	0.083
HC (cm)	0.429*	0.544*	0.447*	−0.581*	0.400*	0.039
WHR	0.089	0.087	0.062	−0.018	0.116*	0.113*
WHtR	0.365*	0.396*	0.356*	−.0388*	0.364*	0.103
SBP (mmHg)	0.314*	0.352*	0.321*	−0.368*	0.297	0.022
DBP (mmHg)	0.323*	0.297*	0.246*	−0.297*	0.204*	−0.023
FBG (mmol/l)	0.090	0.048	0.189*	−0.321*	−0.009	−0.299*
RBG (mmol/l)	0.057	0.170*	0.071	−0.267*	0.137*	−0.035
Cholesterol (mmol/l)	−0.041	−0.007	−0.031	0.046	−0.075	−0.100
Triglyceride (mmol/l)	0.250*	0.354*	0.259*	−0.346*	0.243*	0.124*
HDL (mmol/l)	−0.176*	−0.09	−0.165*	0.0143*	−0.161*	−0.173
ALT (IU/l)	0.114**	0.207*	0.121**	−0.114	0.110	0.099

When looked at each genders separately similar pattern with a slight improvement in association with females compared to males

* p < 0.01

** p < 0.05

**Fig. 1:**
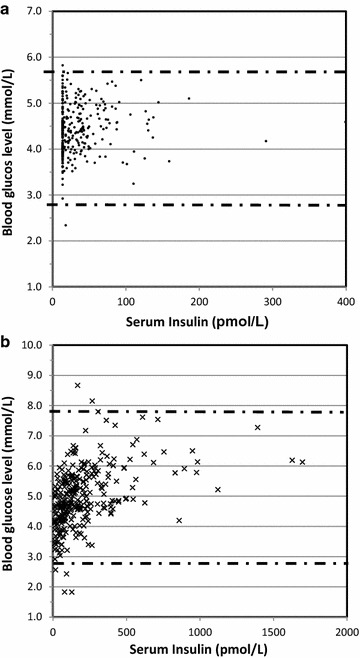
Relationship between insulin and fasting blood glucose (**a**) and insulin 2 h post glucose load (**b**). (The *lines* depict the normal blood glucose levels; a-5.6 and 2.8 mmol/L; b-7.8 and 2.8 mmol/L)

The relationship between blood glucose with concomitant serum insulin was assessed using scatter diagrams (Fig. 1). Scatter plot for fasting blood glucose and fasting insulin showed that the blood glucose levels were managed within normal limits (between 5.6 and 2.8 mmol/L) and with minimum amounts of insulin (Fig. 1a). Apart from a small number of children who had impaired fating glucose, the majority had normal (between 5.6 and 2.8 mmol/L blood glucose levels. However, few required high insulin levels to maintain a normoglycaemic state. When the same plot was drawn for 2 h RBG of OGTT test and insulin levels (Fig. 1b), apart from a few children with impaired glucose tolerance (>7.8 mmol/L), the majority seemed to be well controlled. However, the insulin requirement had been very high in order to maintain normoglycaemia. This denotes that although children had normal blood glucose levels after a glucose load, they had to use large amounts of insulin to achieve it, which shows the strain on pancreas. This could also explain the higher β cell function (HOMA-B) in the groups with deranged metabolic parameters (Table 2).

**Fig. 2:**
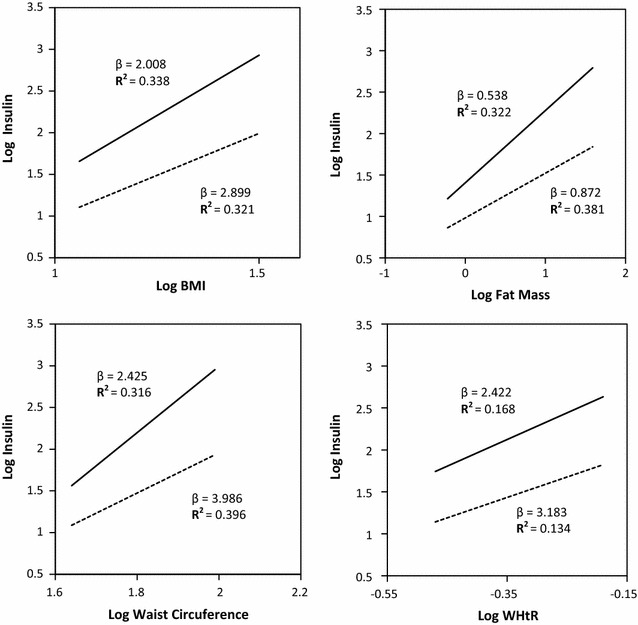
Linear regression to predict log insulin in both fasting and fed states by anthropometric/body composition parameters (interrupted lines fasting state and continued line fed state). Beta coefficient (β) and Coefficient of determination R^2^

Figure 2 shows the relationship between log transformed anthropometric parameters and log transformed insulin levels in both fasting and fed states. In all instances, it clearly showed that with each unit increase in the anthropometric measure of the body, both fasting and post glucose load insulin levels increases significantly. The gradient of the curves were steeper in the post prandial state than in the fasting state.

The sample was divided into nine different groups based on the birth weight tertiles and current BMI tertile values and 3 × 3 tables were constructed (Table 4a–c) for fasting insulin, 2 h post glucose load insulin and HOMA-IR. Metabolic parameters between the three different birth tertiles did not show significant difference when compared within each BMI group. That is between the lowest birth weight and highest birth weight tertile within same BMI tertile.

**Table 4:** Association between different measures of insulin sensitivity and birth and current BMI tertile groups for 5–10 and 10–15 year old age categories

4-a: Mean fasting serum insulin (pmol/l)									
		5–10 years				10–15 years			
		BMI tertile				BMI tertile			
		T1	T2	T3		T1	T2	T3	Total
Birth weight tertile	T1	16.5 ± 8.6	20.5 ± 9.1	33.2 ± 16.6^**^	23.1 ± 13.8	28.5 ± 29.4	35.8 ± 18.7	69.9 ± 34.0^**^	43.9 ± 34.2
	T2	21.9 ± 23.5	26.2 ± 25.6	40.9 ± 28.8	28.4 ± 26.3	19.7 ± 8.5	49.6 ± 92.5	72.6 ± 52.6	46.3 ± 65.2
	T3	14.3 ± 0.9	30.3 ± 33.3	47.6 ± 64.5^*^	32.6 ± 46.2	23.4 ± 16.8	38.9 ± 30.6	56.6 ± 24.4^**^	41.8 ± 28.2
	Total	17.7 ± 14.8	25.6 ± 24.4	40.7 ± 43.3^**^		24.4 ± 21.6	42.3 ± 60.4	66.1 ± 38.2^**^	
4-b: Mean 2 h OGTT serum insulin (pmol/l)									
Birth weight tertile	T1	85.3 ± 73.6	98.1 ± 52.2	250.7 ± 220.4^**^	143.1 ± 154.2	156.7 ± 141.1	261.5 ± 145.3	577.6 ± 465^**^	320.3 ± 343.3
	T2	75.2 ± 60.6	111.5 ± 60.0	211.2 ± 219.8^*^	123.8 ± 130.7	173.8 ± 133.0	241.8 ± 267.9	636.8 ± 567.7^**^	339.8 ± 405.4
	T3	58.9 ± 40.1	67.3 ± 48.3	230.4 ± 199.4^**^	130.2 ± 152.8	118.7 ± 70.2	186.9 ± 141.5	318.3 ± 190.5^**^	217.5 ± 166.2
	Total	75.0 ± 62.7	94.0 ± 56.2	232.9 ± 208.3^**^		154.8 ± 126.0	225.0 ± 199.0	512.5 ± 449.7^**^	
4-c: Mean HOMA IR values									
Birth weight tertile	T3	0.44 ± 0.21	0.57 ± 0.30	0.95 ± 0.51^**^	0.65 ± 0.42	0.81 ± 0.79	0.95 ± 0.46	2.1 ± 1.0^*^	1.2 ± 1.0
	T1	0.56 ± 0.48	0.80 ± 0.92	1.20 ± 0.94	0.82 ± 0.82	0.57 ± 0.27	1.42 ± 2.7	2.2 ± 1.7^*^	1.35 ± 1.94
	T2	0.38 ± 0.05	0.85 ± 1.0	1.29 ± 1.70	0.89 ± 1.3	0.70 ± 0.53	1.2 ± 0.96	1.6 ± 0.71^*^	1.2 ± 0.86
	Total	0.47 ± 0.31	0.74 ± 0.80	1.1 ± 1.2^*^		0.71 ± 0.60	1.22 ± 1.8	1.9 ± 1.2^**^	

Comparison is made across different BMI tertiles within a single birth tertile

When compared between different birth weight tertiles within the same BMI tertile, there were no significance in the difference

Ranges of birth weight tertile distribution are; <10 years, 1.00–2.90, 2.91–3.25, 3.26–4.76 kg; ≥10 years, 1.00–2.80, 2.81–3.20, 3.25–4.25 kg

* Significantly higher (p < 0.05) than the lowest value of the BMI tertiles

** Significantly higher (p < 0.05) than the lowest 2 values of the BMI tertiles

However, when compared between the three BMI groups within one birth weight tertile, those in the highest BMI tertile had significantly impaired metabolic profile than the other two tertiles (Table 4a–c). Those in the lowest birth weight and highest current BMI tertile had the highest insulin secretion in both fasting and fed state and also had high HOMA-IR. Those children in the lowest birth weight and lowest current BMI tertile were spared and behaved similar to children in the other two birth tertiles. This was more marked in older age groups of children. A similar pattern was shown with blood glucose assessed 2 h after glucose load but not with fasting blood glucose, probably denoting the unreliable state of fasting blood glucose assessment (data not shown).

## Discussion

South Asian populations are known to have high insulin resistance [9]. The onset of related changes could be from a young age. Genetic predisposition accompanied with poor intrauterine growth followed by increase growth later in childhood, could probably predispose vulnerable groups to develop insulin resistance later in life.

Our data clearly shows that the insulin secretion in the fasting and fed states are quite high and were similar to the values shown among South Asian migrants living in the UK [21]. It clearly shows that with increase in age and increase in adiposity, higher levels of insulin are required to maintain normoglycaemia. Insulin levels in girls were slightly lower than in boys, which is probably a reflection of early development of insulin resistance in boys resulting in many cardiovascular risks later in life. Many anthropometric parameters showed significant relationship with insulin resistance and were similar to data reported in the region [11]. However, WHR showed a weak association with insulin resistance keeping with some of the past studies in Sri Lankan children [22]. WHR was a poor predictor also in an Indian cohort of children [11], denoting its unsuitability as a predictor of metabolic derangements in South Asian populations. However, WHtR had shown a strong association with insulin resistance. Most of the metabolic parameters showed significant relationships with insulin resistance other than cholesterol. Fasting Insulin showed a weak association with most of the metabolic parameters, which was quite similar to the distribution seen among post-pubertal Indian children [11]. When insulin resistant markers were evaluated between the normal and deranged metabolic parameters, they were significantly higher in the abnormal parameter, as seen with other children of South Asian origin [11]. This shows there is a clear relationship between the development of insulin resistance and derangement of metabolic parameters. However, insulin resistant markers did not show any difference between normal and elevated cholesterol levels.

High insulin levels in the light of normoglycaemia indicated that blood sugar levels, whether in fasting or fed state, are not suitable for detecting cardio vascular derangements at an early stage. The elevated insulin levels with normoglycaemia eloquently describes how insulin resistance precedes the development of dysglycaemia. Therefore, the use of insulin or HOMA-IR as a screening tool for early detection of insulin resistance is highlighted by this study. McAuley et al. have suggested fasting insulin and triglycerides as simple screening tools to detect insulin resistance in the population [23]. Viner et al. suggested screening children with obesity irrespective of ethnicity, age and pubertal status with both fasting insulin and glucose [24]. A study done on 8 and 11 year old Japanese children showed that HOMA-IR in overweight children was 2.51 ± 1.01 and 46.8% had HOMA-IR value of >2.5. Furthermore, this study showed that fasting insulin and HOMA-IR were significantly higher in overweight children and those with a HOMA-IR >2.5 had higher number of cardiovascular risk factors. This study also highlights that insulin resistance could be seen from a very young age and weight management should start from a very young age [25].

Although HOMA-B is an index of beta cell function and is expected to decrease in diabetes mellitus with deranged metabolic profile, in most of the instances, the HOMA-B was higher in the deranged group than in the normal group (Table 2). This could be due to a change that occurs in the initial stages of the development of insulin resistance where β cells would increase its functional output before function deteriorates.

Many studies have shown that poor intrauterine or early growth has contributed to the development of insulin resistance and other metabolic derangements associated with non- communicable diseases [26, 27]. However, the state of body size as an adult has been overlooked in the evaluation of glucose tolerance to early growth [26]. In the evaluation of blood pressure, it has shown that children born with low birth weight and having a low current weight at 10 years or at 36 years had a blood pressure equal to an individual with normal birth weight and normal body size later in life. However if the low birth weight individual has a body weight in the highest tertile at 10 or 36 years, he/she had a significantly higher blood pressure than those with a low birth weight and is in the lowest body weight tertile at 10 and 36 years of life [26]. Our data as far as insulin resistance is concerned showed a similar pattern; if born in the lowest birth weight tertile and remain in the lowest current BMI tertile, he or she would be spared of having an adverse metabolic profile, but if that person has a higher growth later in life, he/she will then have a significantly adverse insulin resistance profile compared to their counterparts in the lower two BMI tertiles within the same birth weight tertile. This highlights the importance of having a controlled growth within early life especially among those who had been born with low birth weight.

This study highlights the early onset of development of insulin resistance in this cohort of children in Sri Lanka and importance of taking steps early to prevent development of NCD later in life. Although this study represent children from Colombo district it would be important to study a more representative sample from the country and also follow up study beginning from a younger age to assess how adiposity rebound would affect the development of insulin resistance which would be more useful in designing NCD control programmes for Sri Lanka and perhaps South Asian populations.

## Conclusion

Although many children were able to control blood glucose within normal limits, they had very high levels of insulin secretion, denoting that insulin resistance is developing from a very young age, thus highlighting the early onset of development of NCD. Worsening state of adiposity has shown worsen the insulin sensitivity levels. Therefore, it is important to screen obese children for insulin resistance using either fasting insulin or HOMA-IR. Children who were born with a lower birth weight would be at a higher risk of developing insulin resistance later if they have increase rate of growth. Therefore, it is important that how we feed our low birth weight children in order to prevent them developing insulin resistance later in life. Close growth monitoring and maintaining the correct growth velocity would be the key.

## Abbreviations

ALT: alanine amino transferase
BMI: body mass index
DBP: diastolic blood pressure
FSIVGTT: frequently sampled intravenous glucose tolerance test
FBG: fasting blood glucose
FGIR: fasting glucose to fasting insulin ratio
HDL: high density lipoprtein
HOMA-IR: homeostatic model assessment–insulin resistance
HOMA–B: homeostatic model assessment–β cell function
HC: hip circumference
IOTF: International Obesity Task Force
OGTT: oral glucose tolerance test
LDL: low density lipoprotein
RBG: random blood glucose
QUICKI: quantitative insulin sensitivity check index
SBP: systolic blood pressure
TG: triglyceride
WC: waist circumference
WHR: waist hip ration
WHtR: waist to height ratio
